# The relationship between intolerance of uncertainty, coping style, resilience, and anxiety during the COVID-19 relapse in freshmen: A moderated mediation model

**DOI:** 10.3389/fpsyt.2023.1136084

**Published:** 2023-02-14

**Authors:** Ting Wang, Lingwei Jiang, Tiantian Li, Xiaohang Zhang, Sanrong Xiao

**Affiliations:** ^1^Department of Humanities, Jiangxi University of Chinese Medicine, Nanchang, China; ^2^Key Laboratory of Psychology of TCM and Brain Science, Jiangxi Administration of Traditional Chinese Medicine, Jiangxi University of Chinese Medicine, Nanchang, China; ^3^School of Public Policy and Administration, Nanchang University, Nanchang, China

**Keywords:** COVID-19, intolerance of uncertainty, coping style, resilience, anxiety, freshmen

## Abstract

**Background:**

The repeated outbreaks of COVID-19 and the rapid increase in uncertainty have had many negative effects on the public’s mental health, especially on emotional aspects such as anxiety and depression. However, in previous studies, there are few studies exploring the positive factors between uncertainty and anxiety. The innovation of this study is the first to explore the mechanism of coping style and resilience as people’s psychological protective factors between uncertainty and anxiety caused by the COVID-19 pandemic.

**Methods:**

This study explored the relationship between intolerance of uncertainty and anxiety of freshmen with coping style as mediating variable and resilience as moderating variable. A total of 1049 freshmen participated in the study and completed the Intolerance of Uncertainty Scale (IUS-12), Self-rating Anxiety Scale (SAS), Simplified Coping Style Questionnaire (SCSQ), and Connor-Davidson Resilience Scale (CD-RISC).

**Results:**

SAS score of the surveyed students (39.56 ± 10.195) was significantly higher than that of the Normal Chinese score (29.78 ± 10.07, *p* < 0.001). Intolerance of uncertainty was significantly positively correlated with anxiety (β = 0.493, *p* < 0.001). Positive coping style has a significant negative impact on anxiety (β = −0.610, *p* < 0.001), negative coping style has a significant positive impact on anxiety (β = 0.951, *p* < 0.001). Resilience moderates the second half of the influence of negative coping style on anxiety (β = 0.011, *t* = 3.701, *p* < 0.01).

**Conclusion:**

The findings suggest that high levels of intolerance of uncertainty had negative effects mental burden during the COVID-19 pandemic. The knowledge of the mediating role of coping style and the moderating role of resilience may be used by health care workers when consulting freshmen with physical health complaints and psychosomatic disorders.

## 1. Introduction

The COVID-19 pandemic has developed into a global public health emergency. It is highly variable, highly contagious, and most individuals in the population are susceptible (1). According to WHO data, as of December 2022, there have been more than 600 million infections worldwide, including more than 6.6 million deaths. Since the outbreak of COVID-19, the Chinese government and the scientific community have acted swiftly to identify the cause of the disease, while implementing a series of timely and effective measures to contain the spread of the disease. Although the government’s COVID-19 restriction strategy has effectively prevented the spread of the corona virus, it has had a negative impact on people’s mental health, especially long-term closed-off management (2). The COVID-19 pandemic has clearly exposed human vulnerability. It is a historic global health crisis that continues to wreak havoc on millions of lives. Uncertainty and health-related anxieties grow organically in the peri-pandemic and post-pandemic periods. People fear infection, ineffective prevention, inadequate intervention efforts, and uncontrolled viral spread. It is clear that the public is not clear about this (3). We are all asked to cope with the ensuing uncertainty. It has a strong impact on college students, especially the freshmen. They are required to work hard to adapt to the new learning lifestyle, but also to make considerable efforts in managing their mental health (4).

A personality trait caused by negative beliefs about uncertainty and its effects is called intolerance of uncertainty (IU). It may also be an important part in anxiety disorders and depression (5). Freeston et al. first proposed an operational definition: IU is a cognitive, emotional, and behavioral response to ambiguous situations and unknown events. Specifically, the cognitive performance of uncertainty is confusing; emotional reactions include frustration and stress; in behavior, trying to control the future and avoid uncertainty, inhibit uncertainty may lead to behavior. Ladouceur, Gosselin, and Dugas emphasize negative evaluations of uncertainty. Regardless of the probability of an uncertain situation or event occurring, and the consequences, individuals with a high intolerance to uncertainty tend to evaluate it negatively. Dugas, Schwartz, and Francis gave a more pertinent definition on the basis of a comprehensive study of the various concepts. They believe that intolerance of uncertainty is a cognitive bias that perceives, interprets and reacts to uncertain situations or events, which affects individual cognition, emotion and behavioral responses (6). In the face of threats, the lower the tolerance of uncertainty, the easier it is to feel anxious, that is, people who cannot tolerate high uncertainty are more likely to have strong anxiety. Thus, we posit the following hypothesis:

**H1:** Intolerance of uncertainty is positively associated with anxiety.

**FIGURE 1:**
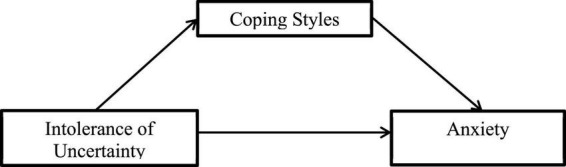
Model hypotheses for mediating coping styles on intolerance of uncertainty and anxiety.

The outbreak of COVID-19 has further increased the level of anxiety among college students. Since 2020, a number of studies have shown that the rate of anxiety among college students in China exceeds 20%, or even more than 40%, of which the cumulative incidence of moderate and severe anxiety exceeds 3% (7, 8). A one-year longitudinal follow-up study showed that during the COVID-19 pandemic, the severity of anxiety among Chinese college students increased significantly, and Sporadic cases still leave college students with a marked increase in anxiety when faced with new cases in their city (9). Although China has already controlled the spread of the epidemic, follow-up studies have found that the anxiety symptoms of college students after the epidemic became normalized were higher than during the initial outbreak (10). For college students, the COVID-19 epidemic is a serious source of stress, and the “unknown/unpredictable sense” it brings has caused great uncertainty to college students. According to the cognitive assessment theory of R. S. Lazarus (11), emotion is the response of individuals to cognition and assessment of the environment. Uncertainty is disgusting, and individual differences that are intolerable to uncertainty affect emotional responses (12). Individuals with high intolerance of uncertainty tend to make a threatening assessment of uncertainty, which leads to fear, anxiety and other aversion reactions (6). Coping is an individual’s cognitive and behavioral efforts to mitigate the negative effects of the environment, while coping styles are the coping strategies that individuals adopt when facing the environment (13). Coping styles can be divided into positive and negative aspects based on their common characteristics. Positive coping styles are more mature and usually include problem solving, help seeking, cognitive adjustment, etc., similar to problem-oriented coping styles, while negative coping styles are relatively immature and include self-blame, avoidance, fantasy, etc., similar to emotion-oriented coping styles (14). The unexpected event of repeated outbreaks of the COVID-19 pandemic creates a high degree of uncertainty, and individuals’ perceptions and opinions of uncertainty influence not only their emotional experiences (15) but also their coping responses to stressors. With the influx of ever-changing and repeated information following the COVID-19 pandemic, individuals who cannot tolerate high levels of uncertainty in the face of these uncertain stimuli often developing negative perceptions and experience negative emotions (16). To cope with these stimuli and the negative emotions and to restore psychological balance, individuals engage in behavioral coping (convergence or avoidance of uncertainty). Furthermore, adaptive outcomes of stress responses vary depending on the coping style. Studies have shown that uncertainty intolerance affects mental health during COVID-19, and coping styles play a mediating role (17). Thus, we posit the following hypothesis:

**H2:** The mediating role of coping style between intolerance of uncertainty and anxiety. The model diagram of our mediation hypothesis is shown in Figure 1.

**FIGURE 2:**
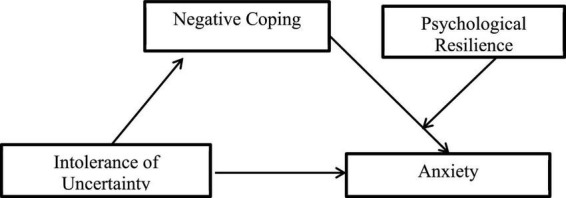
A hypothetical model of psychological resilience to regulate the effects of intolerance of uncertainty on anxiety through coping styles.

Resilience is universal, and it has a protective effect on physical and mental conditions when individuals experience or face adversity (18). Psychological resilience can help individuals after experiencing severe stress or trauma, it allows for good internal control, better adaptation to stressful situations, and a return to pre-crisis conditions to maintain mental health (19). A growing body of literature suggests that resilience helps individuals ward off depression, anxiety, and other negative mental health conditions (20). Psychological resilience may also help mitigate adverse psychological outcomes associated with COVID-19 (21). Research has found that psychological resilience is a protective factor in the psychological impact of the COVID-19 pandemic on people. With the continued impact of the COVID-19 pandemic, especially in the form of health and psychological stress, people in their respective regions need to quickly adapt their thinking and lifestyle to the new changes. Therefore, the role and value of psychological resilience on the physical and mental health status of college students deserves more attention (22). Resilience is defined as positive psychological characteristics that enable individuals to cope effectively with stressful situations. Studies have shown that individuals with high psychological resilience and positive coping styles have lower levels of anxiety and depression during a novel coronavirus pneumonia outbreak (23). Thus, we posit the following hypothesis:

**H3:** Resilience moderated the effect of negative coping style on anxiety. The model diagram of our moderated mediation function hypothesis is shown in Figure 2.

Even before the COVID-19 pandemic, the mental health of college students was a growing concern. The stresses and limitations associated with a pandemic put college students at greater risk for mental health problems, which can severely impact their academic success, social interactions, and future professional and personal opportunities (24). College is a critical period in life, freshman year is the beginning of college life, the personal experiences during this period will affect the growth and development of individuals. Freshmen mainly have developmental and adaptive psychological confusion. Even a few students have serious psychological problems need to be taken seriously. As a special stress group, freshmen are in the transition from parental dependence to independence and from student to socialite (25). Studies have shown that there are significant differences in mental health problems of freshmen in terms of gender, geography, and discipline (26). The study found that the characteristics of freshmen are below: strong herd mentality, simple thinking, strong sense of pride and superiority, unrealistic illusions about college life, strong self-esteem, poor tolerance, and uncertainty of study attitude (27). In summary, as far as we know, Currently, research on individual mental health and coping styles during the COVID-19 epidemic has focused on medical personnel, the general population, and patients with the COVID-19 epidemic (28), there are few studies on the mental health level of freshmen during the repeated COVID-19 pandemic. Therefore, this study focuses on freshmen as the research objective. This study is the first to explore the important buffering effect of intolerance of uncertainty, coping style and resilience on the anxiety of freshmen, and to study the mechanism of intolerance of uncertainty on the anxiety of freshmen, a special pressure group, through positive and negative coping and positive psychological resources (resilience).

## 2. Materials and methods

### 2.1. Participants

A total of 1,049 freshmen participated in the survey. Finally, 1,015 remaining valid data were screened, and the effective recovery rate was 96. 76%. Participants were mainly from two provinces in southern China: 257 from Guangdong Province, 25.3%; 741 from Jiangxi Province, 73%; 17 from other regions, 1.7%. The results showed that 381 were male (37.5%), 634 were female (62.5%); 172 were only child (16.9%), and 843 were non-only child (83.1%); 735 (72.4%) were in the closed-off state, and 280 (27.6%) were in the non-closed off state.

### 2.2. Procedure

Before each participant fills in the questionnaire, they will be informed that the survey is anonymous, and they need to answer all questionnaire items honestly based on their daily life experience. All results will be based on the principle of confidentiality, only for scientific research reference. The authenticity, independence and completeness of all answers are also emphasized. Data acquisition completed in 20 min.

## 3. Measures

### 3.1. Intolerance of uncertainty

Intolerance of uncertainty scale 12 (IUS-12). Developed by Freeston et al. and revised by Buhr and Dugas, consisting of 27 items that assess cognitive, emotional, and behavioral reactions to uncertain situations. It is an assessment tool with a five-item Likert scale (1 = completely inconsistent, 2 = somewhat consistent, 3 = substantially consistent, 4 = very consistent, 5 = completely consistent). The IUS-27 was further simplified by Carleton, Norton and Asmundson into the IUS-12 with 12 items (29). The short version of the Intolerable Uncertainty Scale used in this study was revised by Lijuan Wu in Chinese to form the Chinese version of the short version of the Intolerable Uncertainty Scale (30). It contains three factors: anticipatory behavior, inhibitory behavior, and anticipatory emotion. The final Chinese version of the questionnaire maintains the same items and scoring method as the original questionnaire, with higher scores representing less tolerance of uncertainty, i.e., lower uncertainty tolerance. The revised Chinese version of the IUS-12 has good psychometric properties, with a retest reliability of 0.801. In the present study, the Cronbach’s alpha of the scale was 0.908.

### 3.2. Anxiety

Self-Rating Anxiety Scale (SAS): SAS was compiled by Zung to monitor the anxiety state of patients in the past week (31). The scale includes 20 items, of which questions 5, 9, 13, 17 and 19 are reverse scoring questions, which are scored on a scale of 1 to 4 (1 = rarely to 4 = most of the time). Directly add the scores of 20 questions to form a rough score. Multiply the rough score by 1.25 and take the integer part to get the standard score. The standard score is less than 50 as non-anxiety, 50–59 as mild anxiety, 60–69 as moderate anxiety, and ≥70 as severe anxiety. The higher the score, the higher level of anxiety (32). The Cronbach’α reliability coefficient of the scale is 0.906.

### 3.3. Coping style

The Simple Coping Style Questionnaire (SCSQ) was first developed by Xie and Zhang (33). The questionnaire consists of 20 items and is composed of two subscales: positive and negative coping. Among them, the positive coping subscale consists of 1 to 12 questions, focusing on the characteristics of positive coping; the negative coping subscale consists of 13–20 questions, mainly responding to the characteristics of negative coping (34). The internal consistency coefficient of the questionnaire was 0.90, and the reliability of two dimensions was good: the Cronbach’s alpha coefficient for positive coping was 0.89, and the Cronbach’s alpha coefficient for negative coping was 0.78. The Cronbach’s alpha coefficient of the scale in this study was 0.890.

### 3.4. Psychological resilience

The Psychological Resilience Scale (CD-RISC) consisting of 25 items (35). This scale is the most commonly used scale to measure the “psychological resilience” of individuals in China. It is divided into 3 dimensions, namely optimism, strength and resilience (36). Optimism refers to the individual’s confidence in the development of things and the ability to see things from a positive perspective; strength refers to the individual’s passion and energy for self-improvement in overcoming adversity; and resilience refers to the individual’s perseverance, courage, and strength when under physical or mental stress. The scale is scored on a 5-point scale from “1 = never” to “5 = always.” The higher the score, the higher level of psychological resilience. The internal consistency coefficient and retest reliability of the scale were 0.89 and 0.87, respectively, and both performed well. The Cronbach’s alpha coefficient in this study was 0.953.

## 4. Data analysis

Data analysis was performed using SPSS27.0. There are some reverse scoring questions in the questionnaire design, which are converted before analysis. Harman’s single factor test was used to analyze the variance of the four questionnaires. Descriptive statistical analysis is used to analyze the correlation of population variables. To explore the bivariate correlation between intolerance of uncertainty, anxiety, coping style and resilience, we used independent sample *t*-test, Pearson correlation coefficient. The SPSS PROCESS 4.1 plug-in is then used for mediation analysis (37). Model 4 was used to test the mediating role of positive coping style and negative coping style between the independent variable intolerable uncertainty and the dependent variable anxiety. Then Model 7 and Model 14 were used to test the moderated mediation effect of resilience between intolerance of uncertainty and anxiety on the two coping style paths (38). All tests were within the 95% confidence interval. When the confidence interval did not include zero, the mediating effect was significant at *p* < 0.05.

## 5. Results

### 5.1. Harman factor analysis

In this study, measures such as anonymous answering and reverse scoring of some questions were used to control the common method bias procedurally. The collected data were tested for common method deviation through the Harman factor test. The analysis results showed that a total of 12 factors with eigenvalues greater than 1 were generated, and the maximum factor variance interpretation rate was 18.162% (less than 40%). Therefore, there is no serious common method bias problem in this study.

### 5.2. Demographic data analysis

The results of demographic variables showed that the SAS score of the surveyed students (39.56 ± 10.195) was significantly higher than that of the Normal Chinese score (29.78 ± 10.07, *p* < 0.001). The SAS score of non-only child was significantly higher than that of only child (*t* = 0.335, *p* = < 0.05), and the SAS score of non-normal online shopping was significantly higher than that of normal online shopping (*t* = 3.506, *p* < 0.05). There was significant difference in SAS scores between “whether or not to buy epidemic-related protective equipment” groups (*F* = 4.083, *p* < 0.05). The IU score of the surveyed students was (32.87 ± 9.410). The IU score in the closed-off state was significantly higher than that in the non-closed off state (*t* = 2.294, *p* < 0.05). The score of IU in the high risk group was significantly higher than that in the low risk group (*F* = 2.651, *p* < 0.05). There was a significant difference in the IU score between the “whether to buy epidemic-related protective equipment” groups (*F* = 6.298, *p* < 0.05).

### 5.3. Intolerance of uncertainty, anxiety, coping style, resilience variable analysis

**FIGURE 3:**
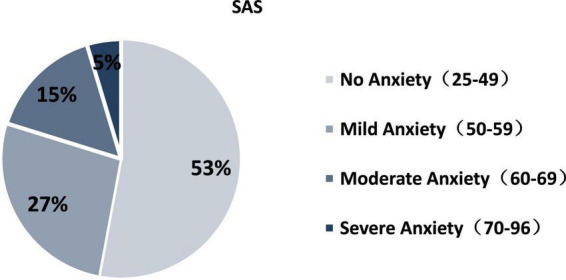
Freshmen’s anxiety self-assessment scores.

Among the samples, 537 had no anxiety symptoms (52.9%), 273 had mild anxiety (26.9%), 158 had moderate anxiety (15.6%), and 47 had severe anxiety (4.6%). As shown in Figure 3.

The descriptive statistics and correlation analysis results of each variable are shown in Table 1. Intolerance of uncertainty was significantly positively correlated with anxiety, significantly negatively correlated with resilience, significantly negatively correlated with positive coping style, and significantly positively correlated with negative coping style. Resilience was positively correlated with coping style and negatively correlated with anxiety. Positive coping style was negatively correlated with anxiety, and negative coping style was positively correlated with anxiety.

**TABLE 1:** Descriptive analysis results.

	M	D	1	2	3	4	5	6	7	8	9	10	11
IU	32.87	9.41	1										
PB	15.59	5.50	0.94**	1									
IB	8.25	2.50	0.72**	0.52**	1								
PE	9.03	2.78	0.87**	0.76**	0.52**	1							
PR	79.98	16.14	−0.07*	−0.21**	0.28**	−0.06	1						
Optimism	12.49	2.91	0.01	−0.10**	0.23**	0.02	0.81**	1					
Strength	26.72	5.48	−0.09**	−0.23**	0.25**	−0.07*	0.94**	0.74**	1				
Tenacity	40.77	8.94	−0.07*	−0.20**	0.27**	−0.08*	0.96**	0.68**	0.85**	1			
PC	34.69	6.51	−0.08*	−0.19**	0.18**	−0.05	0.73**	0.58**	0.71**	0.69**	1		
NC	19.64	4.69	0.27**	0.27**	0.14**	0.24**	0.16**	0.22**	0.09**	0.16**	0.32**	1	
Anxiety	49.45	12.74	0.48**	0.52**	0.22**	0.41**	−0.26**	−0.16**	−0.33**	−0.22**	−0.23**	0.35**	1

IU, intolerance of uncertainty; PB, predictability behavior; IB, inhibitory behavior; PE, predictability emotion; PR, psychological resilience; PC, positive coping; NC, negative coping.

*p < 0.05; **p < 0.01.

**FIGURE 4:**
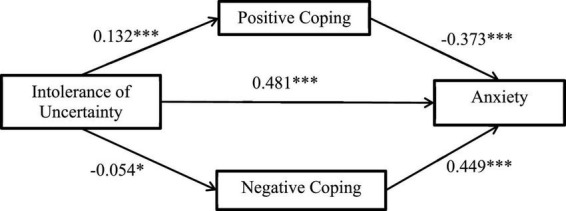
Mediating role of positive/negative coping style between intolerance of uncertainty and anxiety. The results showed that positive coping style can significantly improve anxiety; negative coping style could significantly positively predict anxiety level. *p < 0.05; ^***^p < 0.001.

This study used SPSS extension PROCESS. 4. 1 to test the mediating effect, and the results are shown in Table 2 and Figure 4. With intolerance of uncertainty as the independent variable, anxiety as the dependent variable, and coping style as the mediating variable, Model 4 shows that intolerance of uncertainty has a significant positive impact on college students’ anxiety (β = 0.493, *p* < 0.001). Thus, H1 is supported. Positive coping style has a significant negative impact on anxiety (β = −0.610, *p* < 0.001), negative coping style has a significant positive impact on anxiety (β = 0.951, *p* < 0.001), Thus, H2 is supported.

**TABLE 2:** The mediating role of coping style between intolerance of uncertainty and anxiety.

Outcome variable	Factor	β	SE	t	LLCI	ULCI
Anxiety	IU	0.493	0.036	13.799***	0.423	0.563
	PC	−0.610	0.053	−11.588***	−0.713	−0.507
	NC	0.951	0.076	12.566***	0.802	1.099

IU, intolerance of uncertainty; PC, positive coping; NC, negative coping.

***p < 0.001.

Bootstrap method was used for 5,000 repeated samplings to test the mediating effect of coping style. The results showed that the indirect effect of intolerance of uncertainty on anxiety through coping style did not include 0 in the 95% confidence interval, indicating that the mediating effect of coping style was significant.

**FIGURE 5:**
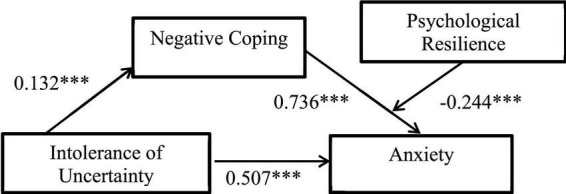
Negative coping style plays a mediating role between intolerance of uncertainty and anxiety. At the same time, psychological resilience can play a regulatory role in the second half of the path. The results showed that negative coping style can increase the level of anxiety, but resilience can significantly inhibit this effect. ^***^p < 0.001.

Model 14 was used to test the moderating effect of psychological resilience on the second half of anxiety through coping style, the results are shown in Table 3 and Figure 5. The product of negative coping style and resilience had a significant predictive effect on anxiety (β = 0.011, *t* = 3.701, *p* < 0.01), indicating that resilience played a moderating role in the prediction of anxiety by negative coping style. The confidence interval of the model test does not contain 0, indicating that the moderated mediating effect is significant. Thus, H3 is supported. That is to say, the coping style of college students with low level of resilience has a greater impact on anxiety. In summary, the moderated mediation model proposed in this study has been supported by empirical data. Coping style plays a mediating role between intolerance of uncertainty and anxiety, and the second half of the mediating role of negative coping style is regulated by psychological resilience.

**TABLE 3:** The moderating effect of psychological resilience on the influence of coping style on anxiety.

Outcome variable	Factor	β	SE	t	LLCI	ULCI
Anxiety	NC	0.736	0.073	10.131**	0.593	0.878
	R	−0.244	0.021	−11.848**	−0.284	−0.203
	NC × R	0.011	0.003	3.701**	0.005	0.017
	Sex	−1.747	0.661	−2.642**	−3.045	−0.449

PC, positive coping; NC, negative coping.

**p < 0.01.

## 6. Discussion

This study was the first to investigate factors associated with negative emotions in freshmen in the context of recurrent outbreaks of the COVID-19 pandemic, exploring the relationship between intolerance of uncertainty, psychological resilience, coping styles, and anxiety. It is also the first study to focus on the mediating role of coping styles between IU and anxiety in freshmen. The details were as follows: freshmen had higher SAS scores than the normal Chinese; IU was positively related to anxiety; coping styles partially mediated the relationship between IU and anxiety; and psychological resilience moderated the effect of negative coping styles on anxiety.

The results show that IU can positively predict individual anxiety level, which verifies our hypothesis 1. The concept of sensitivity to uncertainty has always been considered as an evolutionary adaptive protection factor. In the course of human psychological development, most people learn to be more tolerant of uncertainty, but if they fail to do so, they are more likely to become or remain anxious (39). According to the cognitive model of anxiety, individual perception and evaluation of threat information will affect the generation of anxiety (40). Many studies have linked intolerance of uncertainty to anxiety, and found that intolerance of uncertainty can predict the level of anxiety (41). Chen et al. (42) found that intolerance of uncertainty is an important cognitive risk factor for anxiety and related symptoms. Therefore, this result is also consistent with previous studies (43). According to demographic data analysis, individuals in high-risk areas, in a closed-off state, and unable to shop online normally have shown a high level of intolerance of uncertainty. These performances precisely reflect the cognitive characteristics of college students in the face of uncertain events: the epidemic repeatedly mixed with overwhelming information, and the immaturity of their thoughts makes them more sensitive to the dangerous signals revealed by uncertain events (44), and it is easier to classify fuzzy information as dangerous signals (45). This cognitive bias in the face of serious public health emergencies makes people more likely to produce irrational beliefs and negative emotions (46). Barlow DH. Studies have found that when individuals often experience uncontrollable, they usually cause serious emotional distress and even anxiety disorders (47). This study also supports this result.

The study of Nicholas suggests IU may serve as an important transdiagnostic feature across anxiety disorders and depression (48). This reminds us that when preventing and intervening in college students’ mental health problems during the COVID-19 pandemic, on the one hand, we can cultivate and train college students’ uncertainty tolerance, on the other hand, we should pay attention to improving college students’ emotional regulation ability (49), science emotional theory knowledge and effective regulation methods, so as to enhance their cognition and relief of their negative emotions (anxiety).

The study found that coping style plays a mediating role between intolerance of uncertainty and anxiety. Individuals with low tolerance of uncertainty are prone to psychologically exaggerate the expected possibility and severity of disasters in the face of stressful events or adversity (50). They are more inclined to think they can’t cope with the situation, which creates a higher level of anxiety (51), hypothesis 2 is verified of this study. There was no significant difference in coping style between genders. Zhang et al. (52) conducted a survey of 660 college students in Beijing and found that there were significant differences in stress coping styles between only children and non-only children, and between male and female college students. Liu Chunyan and Li Wenquan conducted a study of 204 normal university students and found that when college students face stress, there are significant gender differences in negative coping styles (53). The results of this study are inconsistent with those of the predecessors, which may be due to the suddenness and severity of COVID-19, as well as the large degree of unknowns and sense of lack of control over it, requiring university students to mobilize all internal and external resources to deal with the negative effects of this major public health event (54). Meanwhile, college students are at the stage of transition from dependence on parents to independence and from students to social beings, therefore, both the demands of the environment and the need for self-growth make them choose positive ways to cope, integrate into college life as soon as possible, adapt to the study environment and interpersonal environment of college, and get into the right track of study (55). This may also be due to the fact that with economic development and social progress, the requirements and expectations of society and families for boys and girls are gradually aligned. As a result, both boys and girls are able to cope well with various problems without significant differences (56). But there was significant difference in the frequency of purchasing epidemic protective equipment. Participants who regularly purchased epidemic protection products (such as masks, alcohol sprays, lotus qingwen capsules, etc.) scored higher on positive coping styles. There is a positive correlation between negative coping and anxiety, which is consistent with the results of previous studies. This result is consistent with previous studies (57). The more individuals tend to use positive coping styles, such as cognitive reappraisal, problem solving, and seeking help, the less psychological problems (58); on the contrary, the more obvious the individual negative coping style, such as the more individuals tend to use avoidance coping style, the more negative emotional response, the greater the degree of anxiety (59). In public health emergencies, the anxiety of college students with positive coping style tendency will be reduced, while the negative coping tendency will aggravate the degree of anxiety (60). Therefore, the mediating effect of coping style is significant, which is also consistent with the previous research results (61). This may be because the epidemic situation changes rapidly and the situation is changing. Freshmen have poor tolerance. When they cannot tolerate uncertainty, it will cause different levels of anxiety, and individual coping styles will indirectly affect anxiety.

In this study, there are significant gender differences in the level of resilience. Cheng et al. showed in the ‘National Sampling Survey Report on Resilience of Chinese Adults’ that there are gender differences in the average scores of resilience dimensions, which is similar to the results of this study (18). There was a significant correlation between resilience and unbearable uncertainty, different coping styles and anxiety level, and it was statistically significant in regression analysis, which was similar to the results of related studies (62). In order to further explore the mechanism of action between IU, anxiety, resilience and coping style, a moderated mediation model test was conducted, and it was found that psychological resilience can regulate the second half of the impact of negative coping styles on anxiety. This suggests that when individuals face repeated outbreaks and cannot tolerate uncertainty, adopting a negative coping style exacerbates anxiety, and individuals with high levels of resilience weaken this effect. Resilience theory suggests that resilience not only protects individuals in adverse circumstances, but also allows individuals who have already suffered danger and trauma to recover from negative events (63); Resilience, as a protective factor, has been shown to appropriately reduce the association between risk factors in life and depression, which can effectively buffer negative outcomes such as anxiety, depression and post-traumatic stress disorder (64). Therefore, based on the previous theoretical basis and the data support of this study, Hypothesis 3 is supported. The results suggest that attention should be paid to the training of freshmen’s problem-solving strategies and skills to enhance their positive coping tendency (65). In the event of a major public health emergency, in the face of various uncertainties, psychological education and psychological training should be increased, which can effectively improve coping styles and psychological resilience (66). Therefore, by increasing psychological training to improve the psychological function of freshmen can be used as a way to solve emotional problems such as anxiety.

In summary, during the recurrence of the COVID-19 pandemic, freshmen generally have a high level of intolerance of uncertainty, a poor level of psychological resilience, and a high level of anxiety. When constructing a psychological intervention system, colleges and universities should focus on factors such as gender and whether they are in a state of containment, give full play to the protective role of psychological resilience, promote students to adopt positive coping styles to face and deal with the uncertainty caused by serious public health emergencies, reduce the generation of negative emotions and maintain normal psychological function (67).

## 7. Limitations

The above discussion complements the anxiety status of freshmen caused by the repeated period of COVID-19, and demonstrates its psychological mechanism with empirical research, but there are still the following deficiencies: First of all, in theory, although the research has successfully proved the mediating effect of coping style on IU and anxiety, the participants are not representative enough. All participants selected in our study are freshmen, and their promotion in other groups is insufficient. Secondly, the focus of this study on the mediating and moderating effects between IU and anxiety is mainly on coping style and resilience. There are other variables in reality, such as risk perception, fear of COVID-19, etc., which need to be further studied. Thirdly, this study adopts a cross-sectional design research method, which cannot accurately determine the causal relationship in the study. Future research should use experimental or tracking research to better design and investigate. Fourth, like many self-reported data collection studies, the participants in this study may also have a social approval effect when answering questions. Future studies may consider a more rigorous design to arrive at more generally efficient conclusions. Finally, this study was conducted in a sample of Chinese college students, which may have cross-cultural inconsistencies, suggesting that similar studies can be conducted in other types of samples in the future.

## 8. Significance

Despite these limitations, this study is the first to explore the internal connections and mechanisms among IU, anxiety, coping styles, and resilience in the context of Chinese culture, taking freshmen as the research objective.

## 9. Conclusion

1. Intolerance of uncertainty is positively associated with anxiety.
2. The mediating role of coping style between intolerance of uncertainty and anxiety.
3. Resilience moderated the effect of negative coping style on anxiety.

## Data availability statement

The original contributions presented in this study are included in the article/supplementary material, further inquiries can be directed to the corresponding author.

## Ethics statement

The studies involving human participants were reviewed and approved by Research Ethics Committee of Jiangxi University of Chinese Medicine. The patients/participants provided their written informed consent to participate in this study.

## Author contributions

TW and SX: conceptualization, methodology, validation, writing—review and editing, and supervision. LJ, TL, and XZ: investigation and writing—original draft preparation. SX, LJ, and TL: data analysis and models conceptualization. All authors have seen, wrote, approved the manuscript, and revised the manuscript.
